# Small Intestinal Polyp Burden in Pediatric Peutz–Jeghers Syndrome Assessed through Capsule Endoscopy: A Longitudinal Study

**DOI:** 10.3390/children10101680

**Published:** 2023-10-12

**Authors:** Jeremy Stewart, Nathan R. Fleishman, Vincent S. Staggs, Mike Thomson, Nicole Stoecklein, Caitlin E. Lawson, Michael P. Washburn, Shahid Umar, Thomas M. Attard

**Affiliations:** 1 Division of Pediatric Gastroenterology, University of Texas Southwestern Medical Center, Children’s Medical Center, Dallas, TX 75235, USA; 2 Division of Gastroenterology, Levine Children’s Hospital, Charlotte, NC 28203, USA; 3 Biostatistics and Epidemiology Core, Division of Health Services and Outcomes Research, Children’s Mercy Hospital, Kansas City, MO 64108, USA; 4 Department of Paediatric Gastroenterology, Sheffield Children’s Hospital NHS Foundation Trust, Sheffield University, Sheffield S10 2TH, UK; 5 Division of Gastroenterology, Children’s Mercy Hospital, Kansas City, MO 64108, USA; 6 Division of Genetics, Children’s Mercy Hospital, Kansas City, MO 64108, USA; 7 Department of Cancer Biology, The University of Kansas Medical Center, Kansas City, KS 66160, USA; 8 Department of Surgery, The University of Kansas Medical Center, Kansas City, KS 66160, USA; 9 University of Missouri–Kansas City School of Medicine, 2464 Charlotte St, Kansas City, MO 64108, USA

**Keywords:** endoscopy, pediatric disorders, small bowel, polyposis, Peutz–Jeghers syndrome

## Abstract

The management of pediatric Peutz–Jeghers Syndrome (PJS) focuses on the prevention of intussusception complicating small intestinal (SI) polyposis. This hinges on the accurate appraisal of the polyp burden to tailor therapeutic interventions. Video Capsule Endoscopy (VCE) is an established tool to study SI polyps in children, but an in-depth characterization of polyp burden in this population is lacking. **Methods:** We performed a retrospective longitudinal cross-sectional analysis of VCE studies in pediatric PJS patients at our institution (CMKC) from 2010 to 2020. Demographic, clinical, and VCE findings reported by three reviewers in tandem were accrued. Polyp burden variables were modeled as functions of patient and study characteristics using linear mixed models adjusted for clustering. **Results:** The cohort included 15 patients. The total small bowel polyp count and largest polyp size clustered under 30 polyps and <20 mm in size. Luminal occlusion correlated closely with the estimated polyp size. Polyp distribution favored proximal (77%) over distal (66%) small bowel involvement. The adjusted largest polyp size was greater in males. Double Balloon Enteroscopy was associated with a decreased polyp burden. **Conclusions:** The polyp burden in pediatric PJS patients favors the proximal third of the small intestine, with relatively small numbers and a polyp size amenable to resection through enteroscopy. Male gender and older age were related to an increased polyp burden.

## 1. Introduction

Peutz–Jeghers Syndrome (PJS) is an inherited predisposition to small intestinal hamartomatous polyps, starting from early childhood, along with an increased risk of malignancy in diverse organs, mainly in adulthood [1]. The natural history of small intestinal involvement is polyp growth progressing to obstruction through intussusception [2], subsequently surgery, intestinal resection, and potentially, short bowel syndrome with intestinal failure as long-term morbidities [3].

The mainstay of management of PJS in the pediatric population centers around suppression of polyp burden through a variety of options, including device-assisted enteroscopy (DAE) [4], laparoscopic-assisted enteroscopy [5], or surgical enterotomy and resection [6]. The timing and choice of therapeutic modality are based on several factors, including polyp number, size, and distribution (polyp burden); patient age; available resources; and expertise [7]. The accurate determination of polyp burden therefore is a critical aspect of PJS management. Several imaging modalities are available, including radiologic, upper intestinal contrast study, MR and CT enterography, as well as Video Capsule Endoscopy (VCE) [3]. 

There is extensive pediatric experience with VCE; the established indications include gastrointestinal bleeding and small bowel Crohn’s disease [8]. Small intestinal polyps are detected in 8% of VCE in pediatric patients overall, mainly in older children (>10 years age) with PJS [9]. Several studies have reported the use of VCE in adult and pediatric PJS, including favorable comparisons with other radiologic modalities. VCE is superior to small bowel series [10], and it is overall comparable to CTE or MRE. VCE is superior to MRE to detect smaller polyps [11] and amenable to enteroscopy and polypectomy, albeit relatively inferior in determining polyp distribution and accurate sizing of larger polyps. Indeed, VCE is found to be highly reliable in accurately assigning interventional modality in adult patients with PJS [12]. Soares and colleagues reported on 14 adults with PJS undergoing VCE, all with numerous polyps, half of whom had large polyps (>11 mm), most needing DAE, with polyp density being highest in the jejunum [13]. 

The pediatric polyposis literature lacks detailed descriptions of the polyp burden, growth characteristics over time, and impact of therapeutic intervention on polyp burden. Herein, we describe the polyp burden in pediatric Peutz–Jeghers Syndrome (pPJS). Our aims are to describe polyp burden differences by age, including polyp distribution; polyp number; and largest polyp size. Our secondary aims were to study the relationship of estimated polyp size with estimated luminal occlusion and differences in polyp burden associated with device assisted enteroscopy in a cohort of pediatric patients with Peutz–Jeghers Syndrome.

## 2. Materials and Methods

### 2.1. Ethical Conduct of Research 

Review and approval were granted by our institutional research integrity body (Children’s Mercy Institutional Review Board Study #00001539 effective 10 May 2020). Informed consent was waived as it was not applicable as per the IRB determination. 

### 2.2. Cohort Accrual 

We performed a retrospective analysis of all VCE studies (Pillcam^®^ SB2, SB3, Medtronic, Minneapolis, MN, USA) at our institution (1/2010–12/2020) in pediatric patients (≤21 years age) with the clinical diagnosis of PJS based on standard criteria as described in the pediatric guidelines factoring hamartomatous polyp number, presence of characteristic mucocutaneous freckling, and family history [14]. 

**Figure 1:**
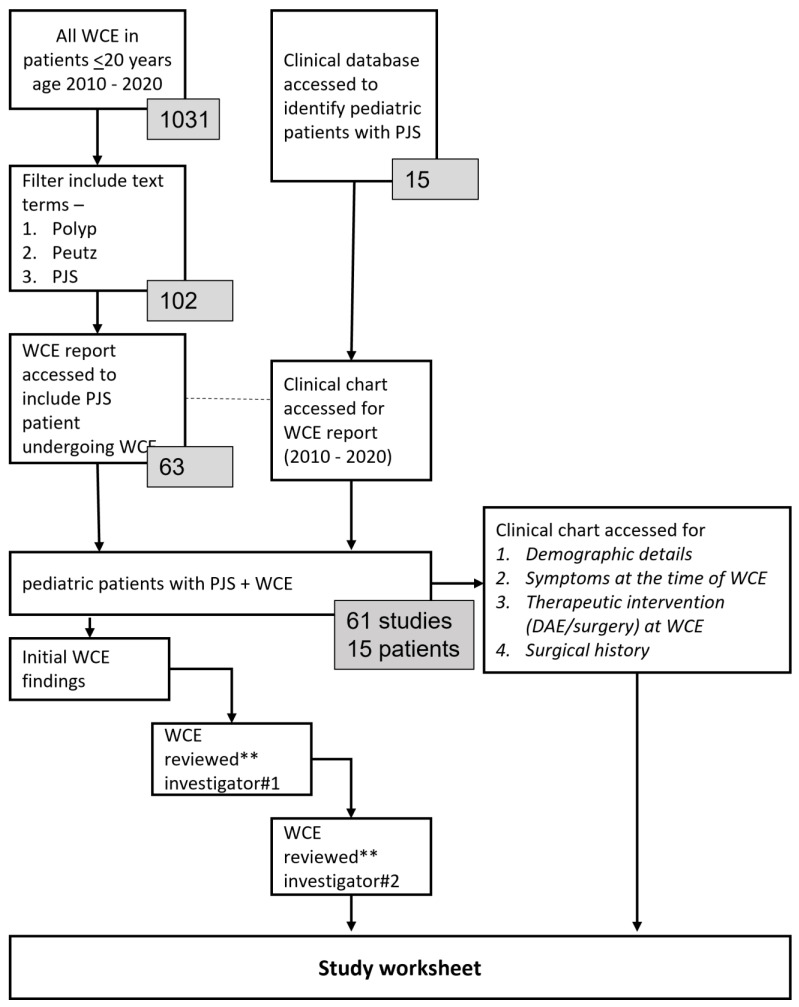
Cohort accrual (methods). ** serial review/report by initial (non-study) clinician reviewer, NF or JS, followed by TMA.

Patients were accrued through interrogation of our pediatric VCE clinical database for the search terms polyp, Peutz(–Jeghers), and PJS, then cross referenced for additional patient accrual with our Pediatric Hereditary Polyposis Registry (Figure 1).

### 2.3. Individual Data Extraction

The accrued study reports were accessed to determine suitability for inclusion. Identified subjects were included in a study database including additional clinical datapoints obtained through clinical chart review. Abstracted details included demographic data, including gender assigned at birth, and age at the time of reported study. Clinical information included symptoms at the time of study and relationship of the study to therapeutic intervention, including DAE or surgery. The individual studies were then accessed by the authors who are experienced endoscopists (JS, NF in tandem with TA) so that each study was reviewed three times (including the initial clinical review) to maximize polyp detection. Studies were reviewed in normal light at frame speed 20–24.

### 2.4. Data Recording

Studies were categorized as complete (capsule reached cecum) or incomplete (last recorded image in the small intestine or indeterminate). Studies were further categorized by the reviewer by the quality of cleanout or presence of artifacts as poor, fair, good, or excellent. Observations were recorded in a study worksheet in categories including polyp number; polyp size expressed as estimated largest diameter (cm); luminal occlusion expressed as a visual estimate of lumen taken up by polyp tissue; and polyp distribution categorized by tertile as determined by the Rapid Reader^®^ package, PillCam (TM) Reader Software v9.0. Incomplete studies were not included in observations of polyp distribution by tertile. Instances of substantive disagreement between reviewers were addressed by review of the original video. The report was revised, if needed, by a more experienced endoscopist (TMA). Disagreements were not recorded or analyzed as part of the study.

### 2.5. Statistical Analysis

We explored total polyp count, maximum polyp size, and maximum luminal occlusion as outcome variables. Total polyp counts and polyp counts within each tertile of the small intestine were grouped in ordered categories; raw counts were not recorded. These categories were numbered as follows for analysis: 0 (0 polyps); 1 (1–10 polyps); 2 (11–20 polyps); 3 (21–30 polyps); 4 (31–40 polyps); 5 (41–50 polyps); 6 (51–60 polyps); and 7 (61–70 polyps). The maximum polyp size was categorized and coded as follows: 0 (no visible polyps); 1 (<0.5 cm); 2 (0.5–1.0 cm); 3 (>1.0–2.0 cm); 4 (>2.0–3.0 cm) and 5 (>3.0 cm). The maximum luminal occlusion was coded as 0 (no occlusion); 1 (occlusion < 12.5%); 2 (12.5–25.0%); 3 (25–50%); 4 (50–75%); or 5 (>75%).

Bivariate associations between these outcome variables and six explanatory variables were estimated by fitting a series of linear mixed models. We chose this approach over an ordinary correlation matrix because the standard assumptions of independent (not clustered) observations and normally distributed, homoscedastic error terms were not met. Patient age at procedure, gender, and symptom status (1 = symptomatic, 0 = not), DBE at visit (1 = yes, 0 = no); surgery visits excluded, total VCEs for the patient during the study period, and study duration. In each mixed model, one of the three outcome variables was modeled as a function of one of the six explanatory variables, with a random patient intercept included to adjust for within-patient clustering. Variables were standardized for modeling. Three additional mixed models were fit to examine bivariate associations in each pair of outcome variables.

In addition, multivariate models were fit to examine each outcome variable as a function of patient gender, age at visit, total VCEs, duration of study visit, and procedure (DBE, surgery, or neither). Again, we fit linear mixed models with a random patient intercept. The polyp count for each tertile was modeled similarly. 

Modeling was carried out using the GLIMMIX Procedure in SAS 9.4, with between-within degrees of freedom specified due to the limited number of patients [15]. Empirical “sandwich” estimators were used as needed to adjust for non-normality and/or heteroscedasticity (assessed by examining plots of model residuals).

## 3. Results

### 3.1. Cohort Characteristics

The cohort included 15 patients (10 male), with a mean age of 12.7 (SD 3.9) years at first included VCE and when diagnosed with PJS. Data were available for a total of 61 VCEs (3.8 VCEs per patient on average). 

All studies were included in the analysis but incomplete studies (capsule did not appear to reach cecum by the end of the study) were excluded from tertile distribution sub-analyses.

### 3.2. Symptoms at the Time of Study

**Figure 2:**
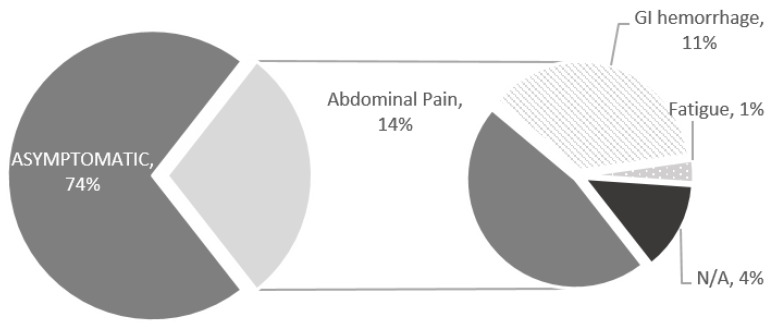
Symptoms at the time of VCE in pediatric PJS.

In most cases (74%), the patient was asymptomatic at the time of VCE (Figure 2). When present, symptoms, could be multiple, included abdominal pain, gastrointestinal hemorrhage, and, relatedly, fatigue. 

### 3.3. Polyp Burden and Distribution

The polyp burden, defined by the total small bowel polyp count and largest polyp size, was clustered in under 30 polyps and <20 mm, with only four studies detecting more than ten polyps and one or more polyps greater than 21 mm in size (Table 1). 

**Figure 3:**
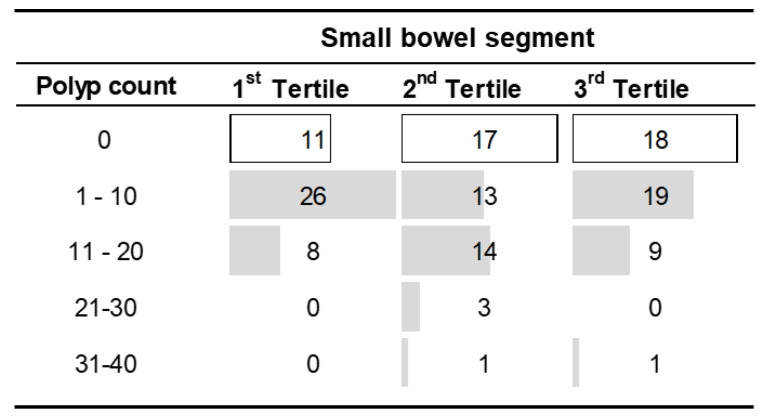
Distribution of polyp counts observed by tertile in VCE (complete studies only).

The polyp distribution (Figure 3) was greatest in the proximal tertiles, with 62% of studies showing non-zero polyp counts in the third tertile in contrast to 76% in the first. The difference, however, related to a greater number of studies showing five or less polyps in the first tertile compared to more distal tertiles.

The total polyp count and maximum polyp size, both grouped into ordered categories, were modeled as a function of patient and visit characteristics using linear mixed models. Models included a random patient intercept to adjust for clustering of visits within patient; between-within degrees of freedom were specified due to the limited number of patients. 

Bivariate associations between the three outcome variables and the set of explanatory variables are reported in Table 2. Higher total polyp counts were associated with a larger maximum polyp size, higher maximum luminal occlusion, and a longer study duration. The maximum polyp size and maximum luminal occlusion were strongly correlated with each other but not with study duration. Females tended to have slightly smaller values for maximum polyp size. There was little evidence that symptomatic patients tended to have a higher polyp burden at the time of VCE.

As shown in the mixed model results in Table 3, the positive association between study duration and total polyp count observed in the bivariate analyses remained after adjustment for the other explanatory variables. The tendency for females to have smaller maximum polyp sizes also remained, with the maximum polyp size for females estimated to be 0.9 categories lower than for males on average. Table 4 illustrates the observations from linear mixed models for polyp count by tertile.

### 3.4. Polyp Burden and Outcome: Relationship with DAE

As part of our clinical protocol to assess residual polyp burden, six patients had 10 VCE studies immediately following Double Balloon Enteroscopy (DBE) with polypectomy. Adjusting for other model variables, polyp counts immediately following DBE procedures were estimated to be 1.0 categories lower (roughly 9–10 fewer polyps) on average than polyp counts for visits with neither DBE nor surgery. The maximum polyp size was an estimated 1.1 categories lower on average for visits involving DBE or surgery than those involving neither DBE nor surgery. Though noteworthy, these effects could not be estimated with enough precision given the sample size to rule out differences in the opposite direction. Interestingly, excluding studies involving surgery, polyp counts in the three highest categories (41–50, 51–60, 61–70) as well as polyp sizes in the maximal luminal occlusion categories (50–75%, >75%) were only observed (13% and 11%, respectively) in VCE studies not following DBE. Furthermore, review of the surgical history in our cohort revealed that six patients had undergone abdominal surgery, including enterotomy with polyp resection; for five of whom this was before the introduction of DBE and for one following the introduction of DBE, but for obstruction from adhesions. 

## 4. Discussion

Surveillance of the small bowel to avoid intussusception and obstruction is the central tenet of preventive management of PJS in children [14]. However, the pattern of small bowel involvement in this population is poorly understood. VCE has emerged as the most reliable method of assessing polyp burden, most notably polyps that are amenable to resection through device-assisted enteroscopy (DAE) including DBE [16,17,18]. This is the largest study to date detailing the polyp characteristics in children with PJS through VCE. Given the importance of expertise [19] and the significant rate of missed lesions upon standard reporting [20], our study maximized polyp detection through in tandem assessment by three credentialed endoscopists and including an experienced capsule endoscopist. 

Gastineau and colleagues retrospectively reviewed 37 VCE reports in 27 children with PJS and compared their findings with different endoscopic studies [21]. As in our study, most patients were asymptomatic; if symptomatic (32%), abdominal pain and anemia were the most frequent symptoms. Similarly, polyp burden did not relate to the presence of symptoms; indeed, asymptomatic patients were more likely to harbor larger polyps (>1 cm). Jejunal polyp distribution was favored over ileal, and both were more frequent than duodenal polyposis. As with our cohort, there were no reported instances of capsule retention. 

In our cohort, most patients were asymptomatic at the time of VCE and when symptomatic, presenting mainly with abdominal pain or gastrointestinal hemorrhage. Symptoms did not correlate with VCE findings, including polyp burden and polyp number, size, or distribution. Accordingly, therapeutic interventions could be correlated with resolution of symptoms but are intended to be preventative and thus relate to our observed decreased need for surgical intervention for obstruction. 

Polyp growth, and therefore polyp size, presumably relative to the lumen, is the principal determinant of risk of intussusception. Polyps with a diameter greater than 20 mm are thought to be at risk of intussusception [22]. Very little is known about the rate of growth of hamartomatous polyps in general and the factors that influence it. There is evidence that the genotype in the patient, specifically truncating mutations of *STK11*, predispose patients to a greater risk of intussusception [23,24]. Our cohort was too small to control for specific genotypic subtypes. Other factors implicated in intussusception risk may include microbiologic factors, but a direct causal association between microbiome factors and polyp size remains elusive [25,26]. In our cohort, a larger polyp size was associated with male gender after adjustment for patient and study characteristics. This may signal an independent factor in polyp growth and therefore intussusception risk. This is a novel observation and suggests that, given the disease expression in the second decade of life and around puberty, hormonal factors may influence small intestinal polyp growth in PJS. There is evidence relating androgen exposure and androgen receptor expression to adenomatous polyp growth [27]. Conversely, estrogen exposure was shown to be protective of adenoma progression [28]. However, we have not encountered any published evidence relating hormonal influences to hamartomatous polyp growth. 

We used two modalities to report polyp size: a size estimate based on visual appearance and an estimate on the percentage luminal occlusion observed with the largest polyp encountered. Estimates of polyp size in PJS based on VCE are reported to be accurate but contingent on the observer’s experience as an endoscopist [19] and with fair interobserver agreement [29]. Earlier attempts to estimate size by comparison to adjacent inert markers have proven unwieldy and have not been broadly accepted [30]. Our estimate of occlusion is based on the maximum area occupied by the polyp tissue as a fraction of the entire lumen and may be easier to reproduce. In our cohort, estimates of polyp size strongly correlated with luminal occlusion. Luminal occlusion may be a more reproducible measure of maximal polyp size, which is in turn the key determinant of the risk of obstruction. Further studies are needed to better characterize this alternative measure of polyp burden. 

The duration of the small bowel viewing portion of the VCE study has been reported to be positively associated with detection of abnormalities, including the source of obscure GI bleeding in adults and small bowel ulceration in children [31]. In our study, the duration of the small bowel viewing portion of the VCE study correlated with total polyp number but not with either estimated polyp size or luminal occlusion. This is somewhat counterintuitive, as the expectation would be that a larger lesion, with more luminal obstruction, would more likely hinder passage of the device. This may therefore represent greater detection of polyps, as the VCE device advances relatively slower through the small bowel or may reflect a polyp influence on intestinal transit. 

None of our studies were preceded by patency capsule ahead of the endoscopic procedure; none of our patients experienced any new obstructive symptoms with the VCE procedure. Granted our small cohort size, this corroborates earlier observations [21] and suggests that routine pre-VCE patency capsule studies are of limited utility in pediatric PJS.

In our center (CMKC), the standard of care is to recommend Double Balloon Enteroscopy based on VCE findings, including larger polyps (>10–15 mm), especially if pedunculated. 

We can infer from our observations that DAE, specifically DBE with polypectomy is an effective strategy in the management of pediatric Peutz–Jeghers Syndrome. This is based on both the clinical outcome observed in our cohort (a reduction in the need for surgery), as well as the observed differences in VCE-assessed polyp burden. VCE studies performed after DBE showed decreased maximal polyp sizes, decreased maximal luminal occlusion and relatively lower polyp numbers. However, post-DBE VCE studies were more likely poor quality. Documenting the residual polyp burden after DAE is an important tenet in management of PJS, as it determines the need for further management and the small intestinal surveillance interval. Despite the logistic advantages of placing a video capsule immediately after DBE, based on our observations, we have moved away from this practice.

Our study has several limitations, including that the VCE studies were spread over 10 years, during which time minor refinements in the performance of the study along with technical improvements in the software and equipment were integrated in our practice. Procedure changes include less aggressive preprocedural bowel cleansing and the routine administration of simethicone to reduce bubbles. Refinements were based on perceived lack of benefit (full prep) or anticipated benefit (simethicone) in reducing artifacts. Software upgrades may have had an impact on the quality of the study, including on the accuracy of polyp detection. As noted, VCE in immediate post-enteroscopy patients was more likely poor quality than other studies, potentially impacting the accuracy of our assessment of the impact of therapeutic intervention. Our study was not designed to compare the detection of polyps by different experienced observers, determine the accuracy of VCE based polyp detection, or provide a comparison with other diagnostic modalities, as these have been addressed previously [11,19,32,33]. 

## 5. Conclusions

This study made several important, including novel, observations. Symptoms were only present in a minority of children with PJS and did not predict polyp burden, polyp number, or maximal polyp size. Polyp burden clustered under less than 31 polyps in total and under 20 mms maximal diameter. Male subjects tended to harbor larger polyps. Polyps tended to cluster in the proximal two-thirds of the small intestine, potentially within reach of upper DBE. The estimated luminal occlusion by polyp tissue was correlated closely with polyp size; further studies are needed to understand its possible use to express risk of obstruction. The duration of VCE studies was related to total polyp number rather than maximal size and there were no instances of polyp retention, questioning the need for routine pre-VCE patency capsule studies. Our findings suggest that VCE-directed DBE is an effective strategy to reduce the polyp burden and decrease the need for surgical intervention in this population. 

## Figures and Tables

**Table 1:** Heat map description of VCE observed polyp number and size distribution in pediatric PJS.

		Maximum Polyp Size (mm)				
Total Polyp Count	No Visible Polyps	<5	6–10	11–20	21–30	>30
0	4	0	0	0	0	0
1–10	0	9	2	1	1	1
11–20	0	3	3	3	1	1
21–30	0	5	6	2	1	0
31–40	0	0	2	1	0	0
41–50	0	1	0	1	0	0
51–60	0	0	0	1	0	0
61–70	0	0	0	1	0	1
NA	0	3	3	1	3	0

(NA—total count not available in incomplete study).

**Table 2:** Bivariate associations: standardized regression coefficients adjusted for clustering.

	Outcome Variable					
Explanatory Variable	Total Polyps		Max Polyp Size		Max Luminal Occlusion	
	Beta (95% CI)	*p*	Beta (95% CI)	*p*	Beta (95% CI)	*p*
Max polyp size	0.5 (0, 0.9)	0.036				
Max luminal occlusion	0.5 (0.1, 0.9)	0.026	0.9 (0.8, 1)	<0.001		
Patient age at procedure	0.3 (−0.2, 0.8)	0.253	−0.1 (−0.7, 0.5)	0.742	0 (−0.6, 0.5)	0.856
Female (vs. male)	0 (−0.4, 0.4)	0.848	−0.3 (−0.7, 0.1)	0.112	−0.2 (−0.6, 0.3)	0.433
Symptomatic	0 (−0.2, 0.2)	0.973	0.1 (−0.2, 0.3)	0.639	0 (−0.3, 0.2)	0.840
DBE (vs. no DBE, surgeries excluded)	−0.1 (−0.4, 0.2)	0.452	0.1 (−0.2, 0.4)	0.560	0.1 (−0.1, 0.3)	0.273
Total VCEs for patient	0 (−0.4, 0.4)	0.930	−0.1 (−0.4, 0.3)	0.610	0 (−0.4, 0.3)	0.812
Study duration	0.4 (0, 0.9)	0.068	0.1 (−0.3, 0.4)	0.609	0.2 (−0.1, 0.4)	0.298

CI: confidence interval, Max: maximum (greatest), DBE: Double Balloon Enteroscopy, VCE: Video Capsule Endoscopy.

**Table 3:** Results of multiple-predictor linear mixed models for total polyp count, maximum polyp size, and max luminal occlusion.

	Total Polyps (Categorized)		Max Polyp Size (Categorized)		Max Luminal Occlusion (Categorized)	
	B (95% CI)	*p*-Value	B (95% CI)	*p*-Value	B (95% CI)	*p*-Value
Female (vs. male)	−0.1 (−1.6, 1.5)	0.945	−0.9 (−2, 0.2)	0.105	−0.5 (−1.9, 0.9)	0.472
Age (years)	0.2 (0, 0.3)	0.044	−0.1 (−0.2, 0)	0.064	−0.1 (−0.2, 0)	0.113
Duration (hours)	0.3 (0.1, 0.5)	0.001	−0.1 (−0.2, 0.1)	0.413	0 (−0.2, 0.1)	0.913
Total VCEs	0.1 (−0.3, 0.5)	0.761	0.1 (−0.1, 0.4)	0.273	0.1 (−0.2, 0.4)	0.393
Procedure						
DBE	−1.0 (−2.5, 0.6)	0.222	−1.1 (−2.7, 0.4)	0.117	0 (−1, 1)	0.955
Surgery	0.2 (−1, 1.4)	0.699	−1.1 (−2.5, 0.4)	0.128	1.3 (−0.9, 3.5)	0.196
Neither	Referent		Referent		Referent	

CI: confidence interval, Max: maximum (greatest), DBE: Double Balloon Enteroscopy, VCE: Video Capsule Endoscopy.

**Table 4:** Results of linear mixed models for polyp count by tertile.

	Tertile 1		Tertile 2		Tertile 3	
	B (95% CI)	*p*-Value	B (95% CI)	*p*-Value	B (95% CI)	*p*-Value
Female (vs. male)	−0.1 (−0.9, 0.6)	0.704	0.1 (−1.3, 1.6)	0.855	−0.1 (−0.7, 0.6)	0.832
Age (years)	0 (−0.1, 0.1)	0.528	0.1 (0, 0.2)	0.070	0.1 (0, 0.1)	0.040
Duration (hours)	0 (−0.2, 0.1)	0.818	0.1 (−0.1, 0.3)	0.149	0.1 (−0.1, 0.2)	0.284
Total VCEs	0 (−0.2, 0.2)	0.885	0 (−0.3, 0.4)	0.826	0 (−0.2, 0.1)	0.888
Procedure						
DBE	0.9 (−0.3, 2.1)	0.102	−0.3 (−1.9, 1.3)	0.657	−0.1 (−1.8, 1.6)	0.880
Surgery	0.5 (−0.3, 1.3)	0.198	−0.4 (−1.5, 0.8)	0.434	−0.1 (−1.5, 1.2)	0.817
Neither	Referent		Referent			

CI: confidence interval, DBE: Double Balloon Enteroscopy, VCE: Video Capsule Endoscopy.

## Data Availability

Data are unavailable due to privacy or ethical restrictions.

## Abbreviations

PJS	Peutz–Jeghers Syndrome
VCE	Video Capsule Endoscopy
CMKC	Children’s Mercy Kansas City
DAE	device-assisted enteroscopy
DBE	Double Balloon Enteroscopy
pPJS	pediatric Peutz–Jeghers Syndrome
MRE	magnetic resonance enterography
PV	pathogenic variant
